# Vibrotactile mapping of the upper extremity: Absolute perceived intensity is location-dependent; perception of relative changes is not

**DOI:** 10.3389/fnins.2022.958415

**Published:** 2022-10-28

**Authors:** Luis A. Pardo, Marko Markovic, Arndt F. Schilling, Meike Annika Wilke, Jennifer Ernst

**Affiliations:** ^1^Department of Trauma Surgery, Orthopaedics and Plastic Surgery, University Medical Center Göttingen, Göttingen, Germany; ^2^Faculty of Life Sciences, Hamburg University of Applied Sciences (HAW), Hamburg, Germany; ^3^Department of Trauma Surgery, Medical School Hannover, Hanover, Germany

**Keywords:** vibrotactile sensation, dermatomes, able-bodies, feedback, psychometric

## Abstract

Vibrotactile sensation is an essential part of the sense of touch. In this study, the localized vibrotactile sensation of the arm-shoulder region was quantified in 10 able-bodied subjects. For this analysis, the six relevant dermatomes (C3-T2) and three segments—the lower arm, the upper arm, and the shoulder region were studied. For psychometric evaluation, tasks resulting in the quantification of sensation threshold, just noticeable difference, Weber fraction, and perception of dynamically changing vibrotactile stimuli were performed. We found that healthy subjects could reliably detect vibration in all tested regions at low amplitude (2–6% of the maximal amplitude of commonly used vibrotactors). The detection threshold was significantly lower in the lower arm than that in the shoulder, as well as ventral in comparison with the dorsal. There were no significant differences in Weber fraction (20%) detectable between the studied locations. A compensatory tracking task resulted in a significantly higher average rectified error in the shoulder than that in the upper arm, while delay and correlation coefficient showed no difference between the regions. Here, we presented a conclusive map of the vibrotactile sense of the healthy upper limb. These data give an overview of the sensory bandwidth that can be achieved with vibrotactile stimulation at the arm and may help in the design of vibrotactile feedback interfaces (displays) for the hand/arm/shoulder-region.

## Introduction

The sense of touch allows us to continuously monitor the boundaries of the whole body. This not only enables safe interaction with our environment even in the absence of the other four main senses but also has a social function when the boundaries of two bodies meet. Consequently, the study of how we perceive the world through somatic sensation has fascinated us for hundreds of years and a lot of data have been generated on this topic (Weber, 1834a, 1851; Weinstein, 1968).

Perception of tactile sensations originates from the receptors located in the skin. These can be divided into four distinct categories: two fast adapting types (FA I and FA II) and two slowly adapting types (SA I and SA II). Type I afferents have small, sharp-bordered receptive fields; type II afferents are larger and have more diffuse borders (Darian-Smith, 2011). The adaption type (slow or fast) indicates the ability to adapt to sustained indentation (Vallbo and Johansson, 1984). The fast-adapting units mainly fulfill vibration sensations. These have two types of end organs: the Meissner corpuscles (FA I), which possess an optimal sensitivity to frequencies of around 50 Hz, and the Pacini corpuscles (FA II), with a peak sensitivity at about 200–300 Hz (Saddik et al., 2011). The Meissner corpuscles are the primary receptors of hands and feet. In contrast, the Pacini corpuscles are mostly found on hairy skin present on the appendages, the trunk, and the head. Various neural roots give rise to the cutaneous nerves that innervate these receptors depending on their site (Johnson, 2001). In the case of the upper limbs and shoulder/ neck region, these roots, located in longitudinal bands around the arm and neck, give rise to seven different dermatomes: Cervical 3 (C3) to Cervical 8 (C8), Thoracic 1 (T1), and Thoracic 2 (T2).

Throughout the upper limbs, the receptor density varies depending on the skin type and the distance from the trunk. Mancini et al. (2014) measured two-point discrimination on the limbs of able-bodied subjects. Their results showed that the minimal distance between the two tips that can be perceived increased from ca. 0.25 cm at the fingertips, to 0.75 cm (the palm), 1.5 cm (the hand dorsum), 2.5 cm (the ventral forearm), and 3 cm (the dorsal shoulder). These results correlate with the findings of Corniani and her group, who estimated an innervation density (in $\frac{units}{cm^{2}}$) of 241 in the fingertips, 58 in the palm, and 13 in the arm (Corniani and Saal, 2020). Koo et al. (2016) studied two-point discrimination on the arm of young Koreans, differentiating between the anterior and posterior parts of the arm. The anterior part of the upper arm is defined as the biceps region and the posterior part as the triceps region. A limitation of this study is that the biceps region is divided between the dermatomes T2 and C5, and the fact that the size of the cortical representation of the dermatomes on the sensory cortex and the number of neurons responsible for interpreting the stimulus differs between dermatomes (Penfield and Boldrey, 1937). Fewer studies address the problem of detecting continuous variation of vibrotactile stimulation. Dideriksen et al. (2021) performed a psychometric evaluation by comparing electro stimulation versus vibrotactile stimulation on the lower arm by varying amplitude and frequency. He showed similar performance of both interfaces at frequencies of 200 Hz, though users are faster at responding to changes in the stimulation parameters during the vibrotactile conditions.

Pathological conditions, such as stroke, brachial plexus injury, cerebral palsy, Parkinson's disease, and amputation, lead to distinct somatosensory dysfunctions. Schneider et al. observed that patients suffering from Parkinson's disease show a significantly higher minimal distances of two-point discrimination than a healthy counterpart on the index finger, but not on the lower arm (Schneider et al., 1987). Tyson described that tactile impairment is more common than proprioceptive impairment after a stroke (Tyson et al., 2008). Similar to amputations in brachial plexus injuries, tactile impairment depends on the level of the injury (Tung and Mackinnon, 2003). Sensory dysfunctions can lead to additional difficulties. For example, Auld et al. showed that spatial tactile deficits account for ~30% of the variance in upper-limb motor function in children with unilateral cerebral palsy (Auld et al., 2012). Furthermore, tactile sensibility plays an essential role in body image and is necessary for experiencing body ownership and pain (Botvinick and Cohen, 1998; Ehrsson et al., 2008; Dietrich et al., 2012).

So far, the analysis of touch mainly focused on its most rudimentary form: short mechanical stimulation. State-of-the-art prosthetics and orthotics, however, often use vibration feedback to communicate several functions, such as switching modalities and velocity among others (Stephens-Fripp et al., 2018a), and the studies including these concentrate on single regions of the arm (Dideriksen et al., 2021). Additionally, little is known about the functionality of named modalities or the requirements for feedback devices at different levels of injury. The necessary size and strength of a vibration device might highly depend on how well the patient perceives vibration on the intended stimulation site. Furthermore, vibrotactile displays are commonly used in a wide array of applications, not only for the upper-limb rehabilitation but also in everyday appliances such as smartwatches and VR headsets (Da-Silva et al., 2018; Orand et al., 2019; Tanaka et al., 2021). Therefore, a proper understanding of how humans perceive vibration is a key to improving these technologies. Most studies investigating vibration so far either focused on the lower arm and hand (Marasco et al., 2018; Stephens-Fripp et al., 2018b; Wilke et al., 2019), on localized spots on some of the main dermatomes (Shah et al., 2019) or predefined arrays on one of the arm regions (Guemann et al., 2019).

A comprehensive mapping of the vibratory tactile sensations in the upper limbs is lacking. To close this knowledge gap, we systematically evaluated the vibrotactile sensations in the lower arm, upper arm, and shoulder region of 10 able-bodied subjects. These included the sensation threshold, the just noticeable difference, and the sensation of dynamically changing stimulation on 12 locations on the arm.

## Methods

### Experimental setup

Ten healthy able-bodied subjects (three females and seven males, all right-handed) participated in the study. All subjects signed an informed consent form approved by the Ethics Committee of the University Medical Center Göttingen (Ethics Number: 26/6/20).

**Figure 1:**
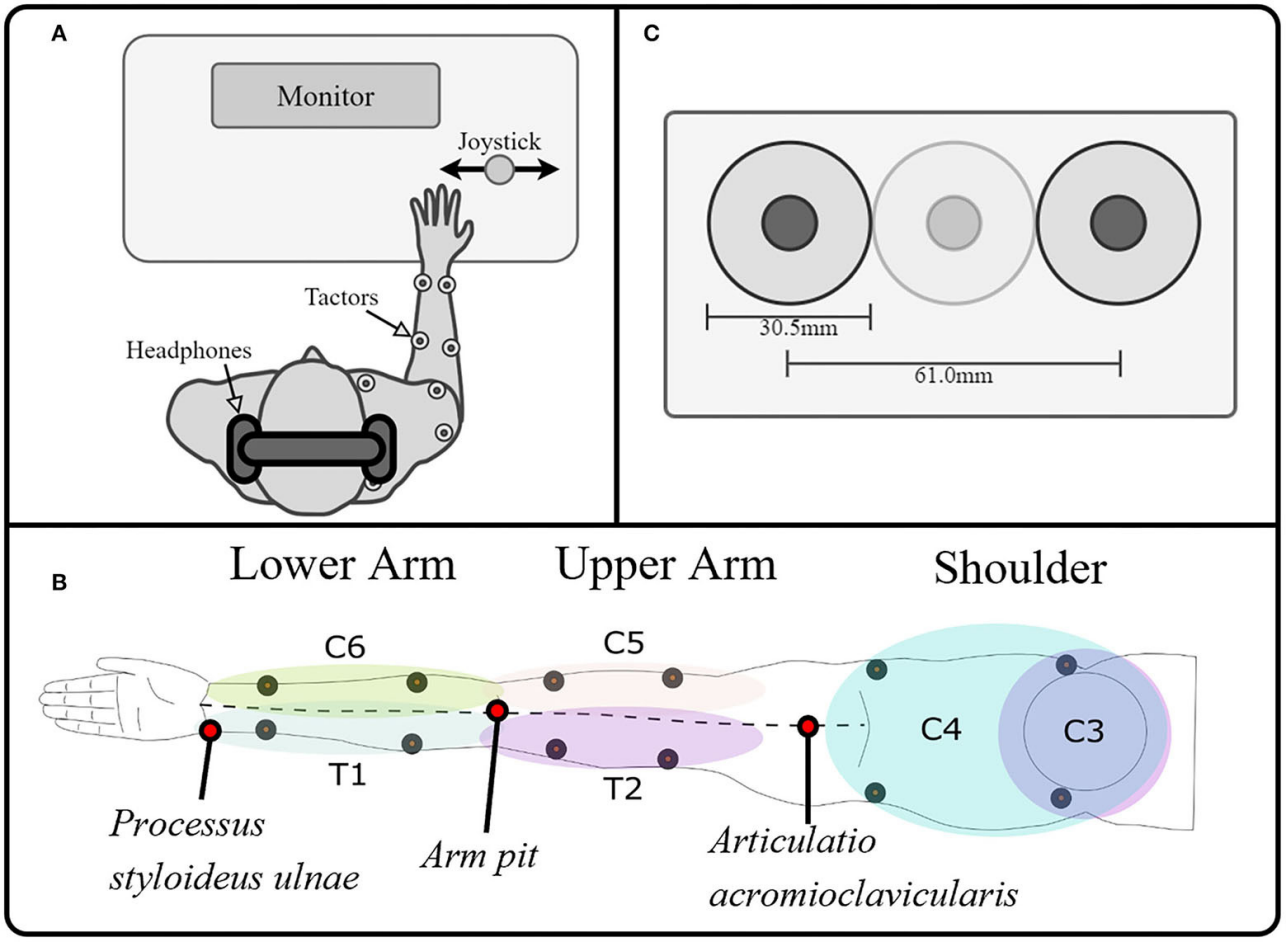
Experimental setup. **(A)** The experimental setup consisted of a PC for data recording, visual instructions, and tactile stimulation; a stimulator to control the tactors; and a joystick as the user interface. The subject was wearing noise-canceling headphones on which white noise was played whenever the tactors were on, to drown the noise generated by the vibration. **(B)**. A total of 12 tactors were placed on the arm and shoulder/ neck, stimulating the dermatomes innervated by the cervical spinal nerves C6, C5 proximally and distally, the thoracic spinal nerves T1 and T2 proximally and distally, and the cervical spinal nerves C3 and C4 ventrally and dorsally, making it possible to map the tactile sensation to vibration over the whole arm. We divided the limb into three segments and evaluated each of them separately. These segments were defined as the lower arm (mainly innervated by T1 and C6), the upper arm (mainly innervated by T2 and C5), and the shoulder (mainly innervated by C3 and C4). **(C)** Additionally, we ensured that two tactors were always separated by at least a tactor-diameter, such that the minimum distance between the vibration centers was at least 61 mm.

We investigated the vibrotactile sensation capacity for each of the six dermatomes of the arm-shoulder region, namely C3, C4, C5, C6, T1, and T2. The tactile sensations were elicited using vibro-tactors placed in pairs of two on each of the dermatomes (Figure 1B). Three types of psychometric evaluations (see sections Experimental tasks and protocol 1–3) were used to quantify the subject's response to the vibrotactile stimulation.

The subjects were seated comfortably in front of a desk with a computer screen during all experimental tasks. They wore active noise-canceling headphones on which white noise was played whenever the tactors were on to prevent the subjects from hearing the vibration of the tactor and ensure that the subject's decision was based solely on tactile sensation (Figure 1A). As a control interface, a modified joystick was connected to a PC *via* a USB port. Its spring was removed to achieve an optimal motion translation with only slight finger movement (HT Series, CH Products, USA). The PC controlled the output of the stimulation channels, which were attached to high-end vibration motors based on voice-coil technology that generate vibrations perpendicular to the skin (C2-tactor, Engineering Acoustics, Inc., USA; diameter: 30.5 mm). These tactors allow, to some extent, separated modulation of frequency and intensity, although the two parameters are coupled through a resonance effect (modulation of the amplitude did not affect the frequency, however, modulation of the frequency will, at some point, affect the amplitude). We controlled the amplitude with a precision of of 0.38% (arbitrary values between 0 and 255, from now on expressed in percentage of the maximal amplitude for easier reference) at their optimal operating frequency of 230 Hz (Engineering Acoustics Inc). We divided the limb into three segments and evaluated each of them separately. These segments were defined as the lower arm (mainly innervated by T1 and C6), the upper arm (mainly innervated by T2 and C5), and the shoulder (mainly innervated by C3 and C4). On each dermatome of these segments, the tactors were positioned using the relevant anatomical features. We measured the length between the *Processus styloideus ulnae* and the armpit (PSU-AP), between the armpit and the *Articulatio acromioclavicularis (APAAC)*, and between *the shoulders (SH)*. We then placed the tactors as follows (Figure 1B):

1. Distal and Proximal T1, C6: $\frac{1}{3}\;\left(PSU-AP\right)$ and $\frac{2}{3}\;\left(PSU-AP\right)$ from the *Processus styloideus ulnae, respectively*.
2. Distal and proximal T2, C5: $\frac{1}{3}\;\left(AP-ACC\right)$ and $\frac{2}{3}\;\left(AP-ACC\right)$ from the *armpit, respectively*.
3. Dorsal C4 and C3: $\frac{1}{3}\;\left(\frac{1}{2}\left(SH\right)\right)$ and $\frac{2}{3}\;\left(\frac{1}{2}\left(SH\right)\right)$ from the *Articulatio acromioclavicularis, respectively*.

- In the ventral part of C4 and C3, the placement had to be slightly adapted, avoiding the clavicle.

Additionally, we ensured that two tactors were always separated by at least a tactor-diameter, such that the minimum distance between the vibration centers was at least 61 mm (Figure 1C). On the lower arm, the tactors were proximal on C6 and T1 because the anatomical distance between distal points (for some subjects) could be lower than the minimally required distance for the simultaneous application of stimuli (i.e., lower than 61 mm). On the upper arm, the distal positions on C5 and T2 were selected to avoid unpleasant sensations induced by a constant vibration near the axilla, where the nervus ulnaris passes superficially. On the shoulder, the ventral locations on C3 and C4 were used because the sensation threshold there was significantly lower than on the dorsal part.

### Experimental tasks and protocol

We performed three experimental tasks to evaluate the vibrotactile sensation capabilities of the lower arm, upper arm, and shoulder. The experimental tasks were carried out in three sessions, one per limb-shoulder region, lasting 1–2 h each with a break of at least 1 hour between the sessions (or the sessions were performed on three separate days). The order of the tested region was pseudo-randomized using all possible combinations (3! = 6). This means, that given the three regions (l – u – s), the list of possible combinations is l – u – s, l – s – u, u – l – s, u – s – l, s – l – u, and s – u – l. In our case, having more than six participants, we simply started the list all over again.

The tasks are summarized here and described below in more detail:

1. We measured the *tactile sensation threshold* by gradually increasing the amplitude of each of the 12 vibro-tactors individually, i.e., quantifying the sensation threshold in four points of each of the arm-regions mentioned above.
2. We calculated the *Weber fraction*, which describes the needed percentual change of the amplitude to be noticeably different for the participant, and the number of distinct intervals that the subject could perceive. This was performed by measuring the *just noticeable difference* in amplitude between two different vibrotactile stimulations delivered on the same spot, one after the other (again in four points per arm region).
3. Finally, we used a *compensatory tracking task* to study the subject's ability to distinguish continuous stimulation on the two main dermatomes of each arm segment. Here, we used an approach called frequency identification of human operators based on the control theory by McRuer and Weir (1969) already used by Dosen et al. (2014) for similar purposes. The human transfer functions obtained in this block allowed us to estimate the human operator's magnitude and phase delay for dynamically changing stimulation signals. This was performed only once per arm region, thus yielding a total of three data points per subject.

#### Sensation threshold

The sensation threshold (ST) was determined using the method of limits on each stimulation side (Botvinick and Cohen, 1998). The experimental task started by selecting one out of four tactors in the selected region from a randomly permutated list that was previously generated with MATLAB's *randperm()* function. This tactor was turned on while the others were kept off. Starting from 0% amplitude, the stimulation intensity of this tactor was increased in steps of 0.78% with a break of 0.5 s between consecutive stimuli. The duration of the stimuli was set to last for 1.25 s. The subject was asked to verbally report the first time (s)he was sure that (s)he perceived the stimulation. After the subject reported that (s)he perceived the stimuli, the stimulation was stopped, and one additional stimulus of 1.25 s duration at the maximum amplitude was applied. The subject was then asked to identify the location on her arm where she perceived the stimulation.

Hereafter, the next tactor in the same region was randomly selected as the active one, and the process was repeated until each of the four tactors was tested three times in each of the three arm-shoulder regions. The overall procedure lasted for about 1 h, and it was performed in one session. Herewith, we obtained the ST for each of the 12 measuring points.

Since the vibro-tactors did not produce painful sensations at the maximum intensity, the upper limit of the intensity range was defined as the maximal stimulation amplitude (100%). Therefore, for each stimulation site *i*, the testable intensity range was defined as *[ST*_*i*_*, 100%], i*=*1, …, 12*.

#### Just noticeable difference

**Figure 2:**
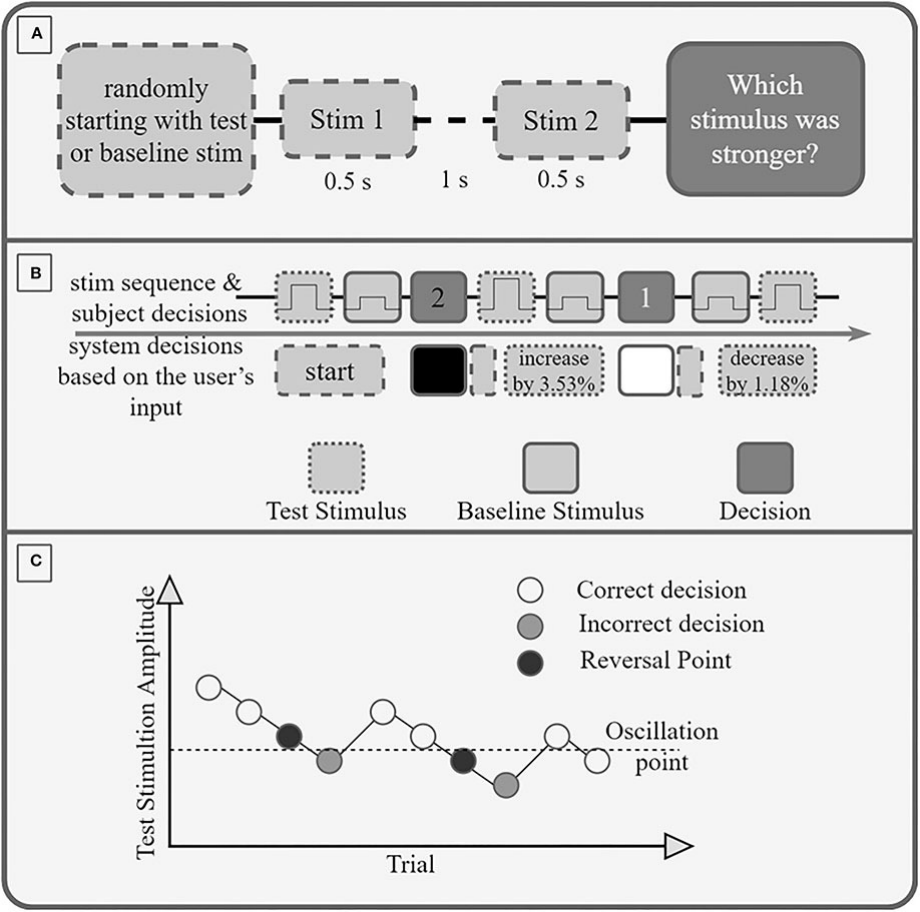
**(A)** Experimental Protocol. If the first stimulus is the test stimulus or the baseline stimulus is decided randomly. Each stimulus is 0.5 s long, and between the stimuli is a break of 1 s. After the second stimulus, the participant must decide (and select *via* the joystick) if the first or second stimulus had a higher amplitude (was stronger). **(B)** Exemplary sequence of trials. A wrong decision increases the amplitude of the test stimulus by 3.53%, a correct decision decreases it by 1.18%. **(C)** Exemplary Staircase sequence. The amplitude of the stimulation is reduced after each correct decision and increased after an incorrect decision. A reversal point is the last correctly recognized amplitude before an incorrect decision. The oscillation point is the average amplitude of 10 reversal points.

During the second task, we measured the just noticeable difference (JND), which described the minimal difference in amplitude between two subsequent stimulations that the subject can perceive. The order of the evaluation of arm-shoulder regions was determined randomly. Once the region was selected, four tactors were placed on the proximal and distal (or ventral and dorsal, in case of the shoulder) sides of the corresponding dermatomes. Like in the previous experimental task, one tactor (i.e., one dermatome) was randomly selected as active. The task continued by stimulating the selected dermatome with two consecutive stimuli of different intensities— a base (lower and constant) and a test (higher and variable) stimulation. The duration of the stimuli was 0.5 s, followed by a break of 1 s before the second stimulus (Figure 2A). The order of the two stimuli was randomized. After each pair of stimuli, the subject was asked to select the stimuli with the higher intensity by turning the joystick left (indicating the first stimulus had higher intensity) or right (meaning the second stimulus had higher intensity). Put differently, the subject was asked to identify which of the two stimuli was the test stimulus. Afterward, the subject was stimulated again, and this process was repeated until a total of 10 reversal points were reached (see below). Within the selected stimulation site, the baseline stimulus always had the same intensity, whereas the intensity of the test stimulus was varied according to the staircase method. Namely, the baseline intensity was fixed to *ST*_*i*_+0.15*(100%−*ST*_*i*_), while the test stimulus was initially set to *ST*_*i*_+0.9*(100%−*ST*_*i*_). Increasing the baseline stimuli by 15% of the perceivable range was performed because we expected that applying stimulation continuously on the same spot would slightly shift the ST upward (due to the adaptation effect), thus rendering the baseline stimuli unperceivable. Likewise, decreasing the first test stimulus to 90% of the testable range was done to prevent overstimulation and thus slow down the overall adaptation to the stimuli. If the subject correctly identified the stimulus with the higher intensity (i.e., the test stimulus), the intensity of the following test stimulus was decreased by 1.18% of the maximum amplitude; if, on the other hand, the subject made a mistake, the intensity of the following test stimulus was increased by 3.53% (Figure 2B).

The oscillation point was identified as the average intensity preceding all stimuli with incorrect identifications across all trials, i.e., as the average of 10 reversal points (Figure 2C).

Finally, the JND of the selected stimulation site was obtained as the difference between the oscillation point (OP) and the baseline intensity expressed as a percentage of the maximum amplitude *JND*_*i*_ = *OP*−(*ST*_*i*_+0.15*(100%−*ST*_*i*_). The overall process was repeated for each stimulation site (i) in each arm-shoulder region, thus yielding 12 distinct measurements of the JND_i_, *i*=*1, …, 12*.

#### Closed loop compensatory tracking

**Figure 3:**
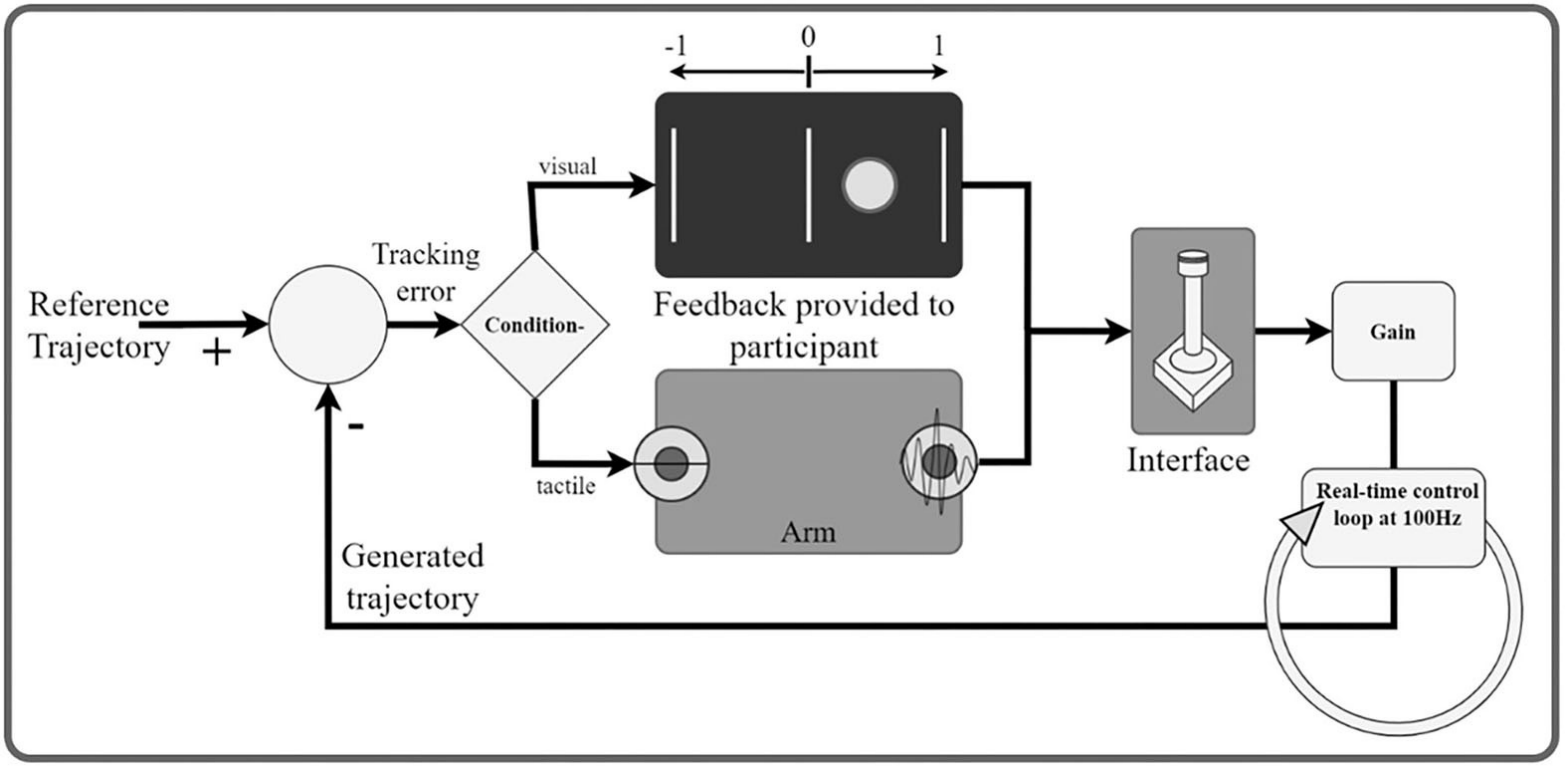
Schematic illustration of the real-time control loop. The participant (human operator) is a part of a dynamic system. The goal is to generate a signal with the joystick that minimizes the tracking error with respect to a predefined reference trajectory. The tracking error is communicated to the participant through tactile/visual feedback. In the visual feedback, the onscreen circle communicates the sign and the magnitude of the error. In the tactile conditions, the active stimulator codes for the sign and the amplitude for the magnitude of the error.

In order to investigate the subject's ability to differentiate between dynamically changing stimulations, we used a compensatory tracking task, in which the subject received two-dimensional information about her performance. More specifically, the subject performed compensatory tracking of a 90 s long reference signal in a real-time control loop by using a joystick as an input interface (Figure 3). The reference signal consisted of a pseudorandom multi-sine wave with nine components between 0.1 and 2 Hz, where the five sine waves with the higher frequencies (>0.4 Hz) had half the power as all other components combined. The signal was in the range of [−1, 1]. The tracking error was defined as the difference between the user input (i.e., the joystick position) and the reference signal. This error was conveyed either *via* two tactile units placed on two different dermatomes on one of the previously selected arm-shoulder regions or *via* the computer screen. To train the task, the subject first performed it using only visual feedback. In this condition, the error was indicated as a red circle on the computer screen, moving along a horizontal axis. In the middle, a green vertical line marked zero error. Therefore, the subject was instructed to move the joystick to keep the red circle on the green vertical line. In the vibrotactile feedback condition, the sign of the error was spatially encoded by the two stimulation units. The stimulation amplitude was proportional to the error magnitude in the range from ST plus 15% of the intensity range (indicating a minimal error of just above zero) to ST plus 90% of the intensity range (indicating the maximum error of one unit; errors higher than one were capped to this value). The sign of error was thereby mapped to a spatial sensation while the intensity of the stimulation was proportional to the magnitude of the error. In the vibrotactile feedback condition, the task for the subject was to minimize the stimulation intensity (zero tracking error = no stimulation). To achieve this, the subject had to move the joystick proportionally to the perceived stimulation intensity and in a direction that is opposite from the active stimulation site. Importantly, although the task was repeated for each arm-shoulder region only one tactor per dermatome was used in the vibrotactile condition.

The subjects performed as many trials as needed in the visual condition to become familiarized with the task (usually one or two attempts). When the subjects reported that they were confident with the task, they were instructed to perform 10 additional trials using visual feedback only. After these 10 trials had been completed, vibrotactile feedback was added to the visual one, and the subject was asked to perform the task three additional times. While performing the task in this visual-tactile condition, the subject was asked to focus on the correlation between the movement of the red circle on the computer screen and the stimulation sensation. Therefore, while performing the task in this condition, the subject could associate the tactile sensation with the visual representation of the tracking error. Finally, the subject proceeded with training to use the tactile feedback only for at least three trials (or more, until (s)he was able to reach a tracking correlation coefficient of 0.6 or above–see data analysis). Afterwards, the actual evaluation started and consisted of 10 additional trials. A break of 1–2 min was introduced after each trial to avoid mental and physical fatigue. As in previous tasks, the order of the evaluated arm-shoulder regions was randomly selected. The overall process resulted in a total of 30 data points for the vibrotactile condition (10 per arm-shoulder region) and the additional 10 data points for the visual condition.

### Data analysis

The following outcomes were used to interpret the acquired data: (1) the sensation threshold at which the participant was able to feel the stimulation, (2) the success rate of identifying the stimulation location (3) the number of distinct intervals (NDI), which refers to the number of intervals separated by the magnitude of the JND within the dynamic range, (4) the Weber fraction, as the needed percentual difference between two stimuli to be successfully identified as different, and (5) the tracking performance interpreted from the delay, the average rectified error, and the correlation coefficient during the tracking task.

For every individual subject, the ST of any of the 12 locations in the arm-shoulder region was estimated by averaging the data obtained from the three ST measurements (trials) that were performed for that location. Then, these values were additionally averaged and compared (1) the three different segments (the lower arm, upper arm, and shoulder) and (2) the two different sides of the arm-shoulder region (the ventral and dorsal).

Similarly, the subject's JND was calculated across individual locations, arm-shoulder segments, and ventral and dorsal sides. In addition, the JND was used to compute the number of distinct intervals (NDI) that a subject could perceive. Since the JND is expressed as a percentage of the maximal stimulation intensity, the NDI was calculated iteratively according to the equation: $I_{k+1}=\;I_{k^{\prime }}+JND*I_{k}$, where *I* indicates the stimulation intensity and *k* counts the intervals. Initially, *k* was assigned the value of one and was increased in steps of one until *I*_*k*+1_ passed the upper limit of the dynamic range (maximum intensity). Once this happened, the NDI was set to the value of *k*. For instance, in a range from 1 to 100 arbitrary units with a JND of 5, there are 20 NDIs. Finally, the Weber fraction (WF) was also calculated from the JND by applying the following formula: $WF_{i}=\frac{JND{\;}_{i}}{b_{i}}*100$ (Weber, 1834b), where *b* indicates the baseline intensity defined in Just noticeable difference.

The outcome measurement of the third experimental task was the trial tracking performance assessed by comparing the shape similarity, average deviation, and time delay between the generated and reference trajectories for each of the three arm-shoulder segments. The correlation between the reference and the generated trajectory was identified as the peak of the cross-correlation function. Furthermore, the time delay between the target and the generated trajectory was estimated from the temporal location of the peak in the cross-correlation. After compensating for this delay, the average rectified error was calculated. Finally, the mean values of these three parameters (shape similarity, average deviation, and delay) were calculated for each subject (and the arm-shoulder segment) by averaging the outcomes of the 10 compensatory tracking trials.

For all outcome measures separately, we performed one-sample Kolmogorov-Smirnov tests and found that none of the outcome parameters was normally distributed. Therefore, we utilized Friedman tests in combination with *post-hoc* Wilcoxon signed-rank tests to detect statistical differences between different locations, segments, and sides of the arm-shoulder region. More precisely, when all 12 locations in the arm-shoulder region were compared, a Friedman test with 12 levels was used. When comparing the three different segments (the lower arm, upper arm, and shoulder), a Friedman test with three levels and hence three *post-hoc* Wilcoxon signed-rank tests were conducted. For the two different sides of the arm-shoulder region (the ventral and dorsal), a single Wilcoxon signed-rank test was sufficient. As none of the outcome measures was normally distributed, only non-parametric statistics and hence one-factor tests could be used.

All statistical tests were corrected for multiple comparisons using the Bonferroni-Holm correction. The statistical difference threshold was set to 0.05. All results are presented as “median (interquartile range (IQR))”.

## Results

To achieve a comprehensive mapping of the tactile sensations in response to vibrotactile stimulation across the whole arm-shoulder region of 10 able-bodied participants, we performed three psychometric evaluations.

### Sensation threshold

**Figure 4:**
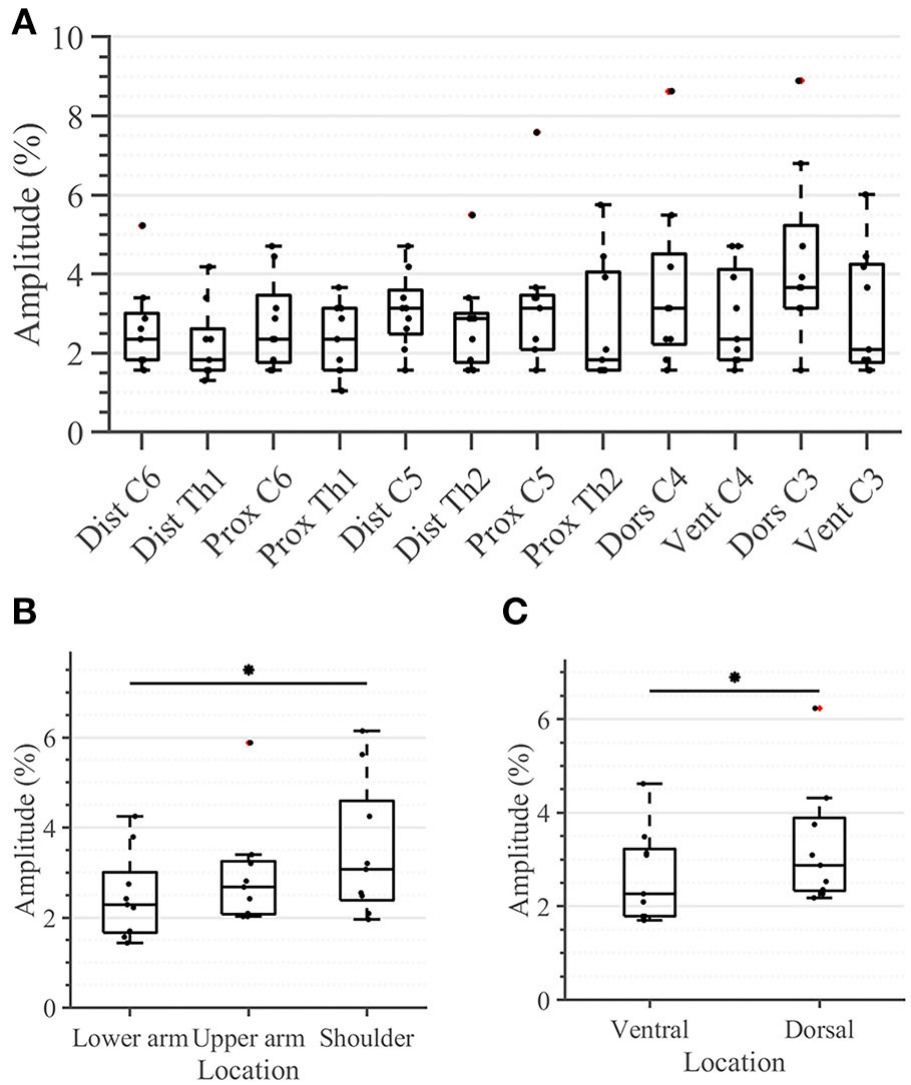
Task 1–Sensation thresholds (in percent of the maximum stimulation amplitude) for **(A)** all 12 locations in the arm-shoulder region, **(B)** the three arm-shoulder segments, and **(C)** the ventral and dorsal side of the arm-shoulder region. The box plots depict the median value (horizontal line) and the IQR (rectangle) of mean ST data collected from 10 subjects. Red crosses indicate statistical outliers within a single box plot (*n* = 10). An asterisk indicates that there is a statistical difference (*p* < 0.05, corrected).

All participants were able to correctly identify the active tactor in all 36 trials (100% success rate; results not depicted). No statistical differences were detected between sensation thresholds (STs) of any of the 12 individual locations in the arm-shoulder region (Figure 4A). However, when the mean ST of the respective four locations on the lower arm, the upper arm, and the shoulder were compared, the shoulder segment exhibited a significantly higher ST than the lower arm (Figure 4B; 3.1 vs. 2.3% (*p* = 0.0039) of the maximal stimulation amplitude). Furthermore, the average ST of the six locations on the dorsal side of the arm was significantly larger than the mean of ST of the locations on its ventral side (Figure 4C; 2.88% in comparison with 2.27% (*p* = 0.0078) of the maximal amplitude).

**Figure 5:**
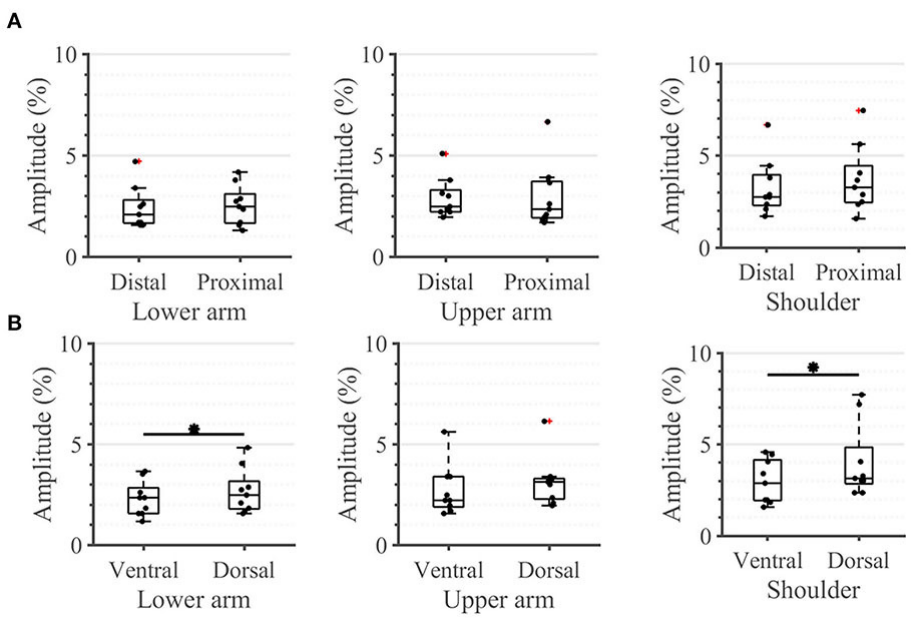
Task 1-Sensation thresholds. A closer look (in percent of the maximum stimulation amplitude) for **(A)** the distal and proximal parts of the three segments of the arm, **(B)** the ventral and dorsal parts of the three segments of the arm. The box plots depict the median value (horizontal line) and the IQR (rectangle) of mean ST data collected from 10 subjects. Red crosses indicate statistical outliers within a single box plot (*n* = 10). An asterisk indicates a significant statistical difference (*p* < 0.05).

Taking a closer look at the single segments, we found no significant differences between the distal and proximal parts of any segment (Figure 5A). Applying the same analysis to the ventral and dorsal area of each segment we identified significant differences in the lower arm and the shoulder (Figure 5B). In both locations, the threshold amplitude on the dorsal side needed to be significantly higher than on the ventral side to detect vibrotactile stimulation (*p* = 0.0391 on the lower arm; *p* = 0.0313 on the shoulder).

### Just noticeable difference

**Figure 6:**
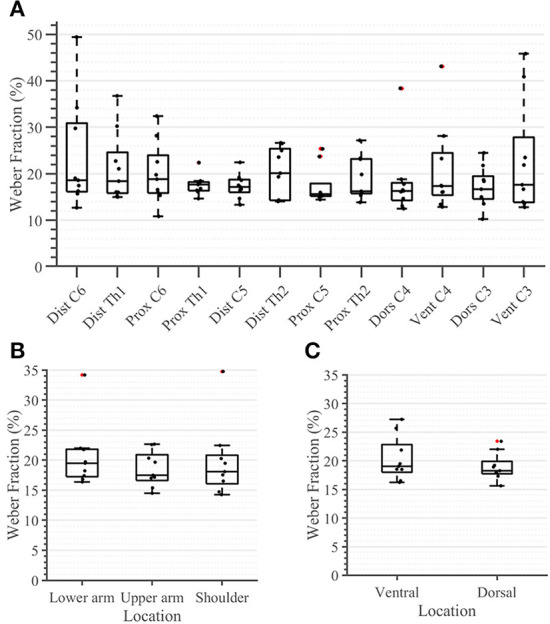
Task 2 -Weber Fraction (in percent) for all 12 locations in the arm-shoulder region **(A)** on the tested parts of the arm, as a combination of the results of the dermatomes in each region (the lower arm, the upper arm, and the shoulder), **(B)** and in each side (the ventral and dorsal), **(C)** (*n* = 10). The red points show outliers.

**Figure 7:**
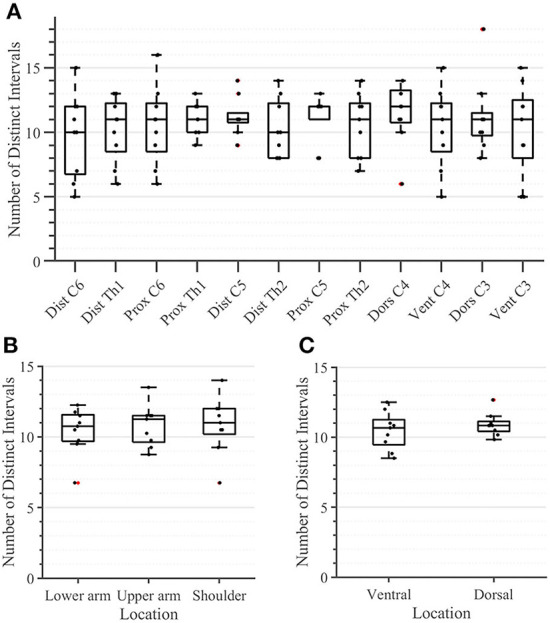
Task 2 -Number of Distinct Intervals for all 12 locations in the arm-shoulder region **(A)** on the tested parts of the arm, as a combination of the results of the dermatomes in each region (the lower arm, the upper arm, and the shoulder), **(B)** and in each side (the ventral and dorsal) **(C)**, (*n* = 10). The red points show outliers.

**Figure 8:**
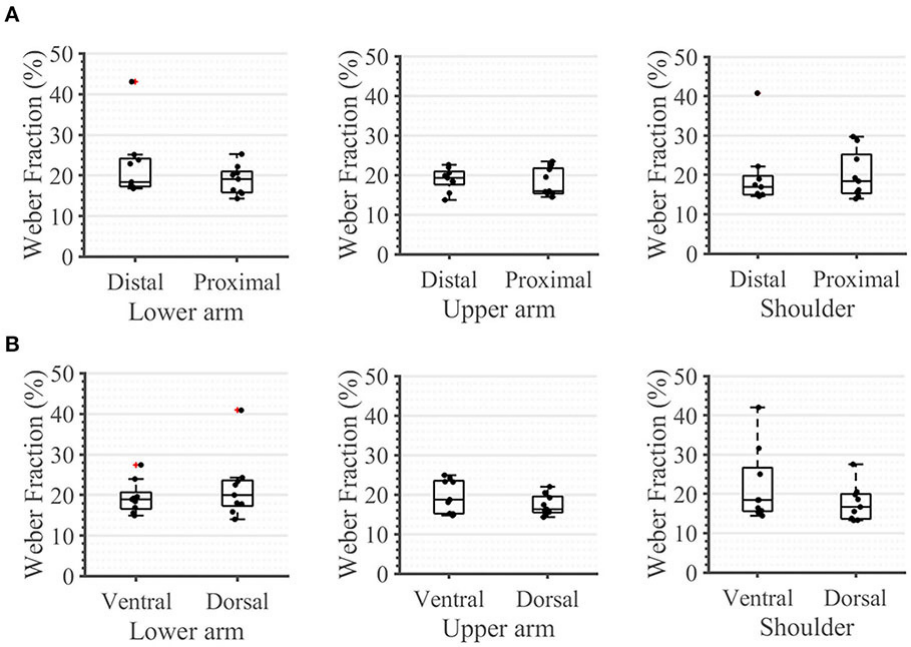
Task 2 -Weber Fraction. A closer look. in percent, for **(A)** the distal and proximal parts of the three segments of the arm, **(B)** the ventral and dorsal parts of the three segments of the arm. The box plots depict the median value (horizontal line) and the IQR (rectangle) of mean ST data collected from 10 subjects. Red crosses indicate statistical outliers within a single box plot (*n* = 10).

**Figure 9:**
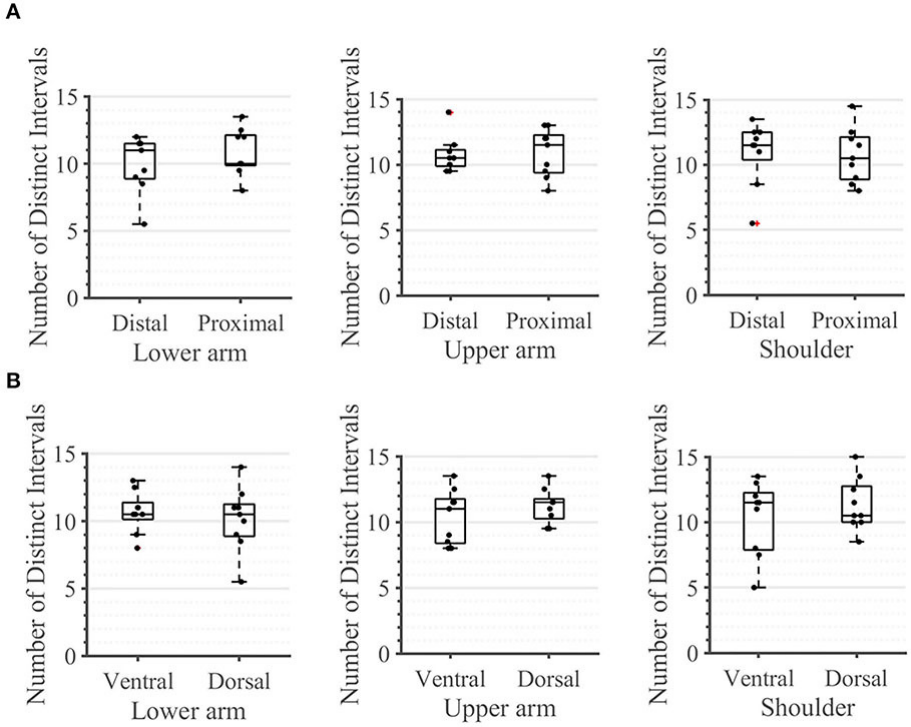
Task 2 -Number of Distinct Intervals. A closer look for **(A)** the distal and proximal parts of the three segments of the arm, **(B)** the ventral and dorsal parts of the three segments of the arm. The box plots depict the median value (horizontal line) and the IQR (rectangle) of mean ST data collected from ten subjects. Red crosses indicate statistical outliers within a single box plot (*n* = 10).

During the second task, the participant had to differentiate between two sequential stimuli and select the one she perceived as having a higher amplitude. Differences between baseline and test stimuli above 20 [7%] were reliably detected across all arm-shoulder regions, without any significant differences between them [neither between single locations (Figure 6A) nor between the defined regions (Figure 6B) or sides (Figure 6C)]. Likewise, the number of discrete steps that could be provided using the obtained tactile sensation range 11 [3%] did not show any significant difference between the arm-shoulder segments (again, neither between single locations (Figure 7A) nor between the defined regions (Figure 7B) or sides (Figure 7C)). Again, a closer look at the single segments exposed neither significant differences between distal and proximal sides nor ventral and dorsal sides of any segment regarding the Weber fraction, and the number of distinct intervals (Figures 8, 9).

### Closed loop compensatory tracking

In the compensatory tracking task, the participant acted as the controller in a closed-loop system, compensating for the error between a generated and a reference signal. (S)he used a joystick as an input interface and received either visual or tactile feedback about her performance. To ensure the subjects understood and adequately performed the task, we used the session in which the subject performed the task receiving visual feedback on the monitor, as a baseline with optimal feedback. The subjects in this condition showed a significantly better performance in all aspects (results not shown).

**Figure 10:**
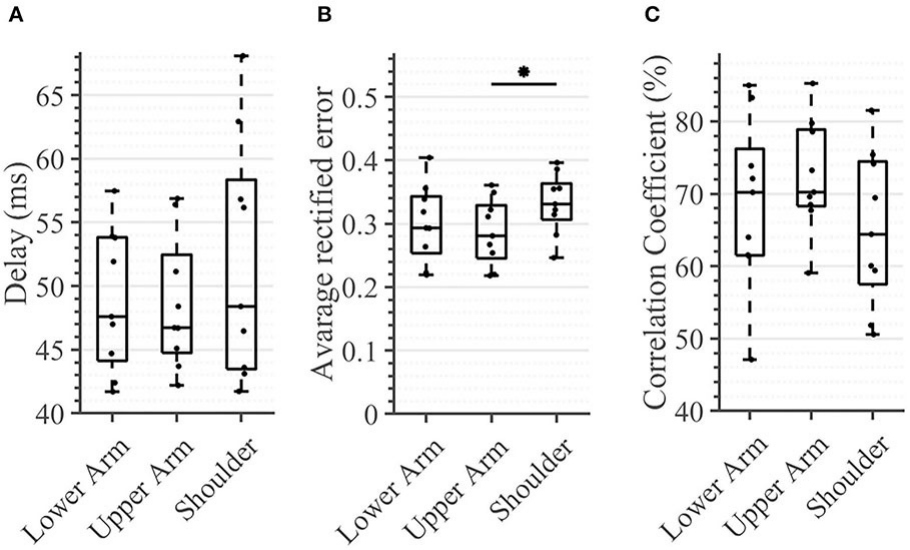
Task 3 -Average performance in the compensatory tracking task for all subjects and conditions. Here, time delay **(A)**, average rectified error **(B)**, and correlation coefficient **(C)** between the target and cursor is shown (*n* = 10). An asterisk indicates that there is a statistical difference (*p* < 0.05).

In the tactile feedback condition, neither the delay nor the correlation coefficient was significantly different between any segments (Figure 10). The delay was consistently below 48.5 ms [11.65 ms] and the correlation coefficient was in the range of 64.4–72.2% [13.6%], for the three evaluated segments. The only detected significant difference was between the shoulder and the upper arm where the average rectified error was significantly higher in the latter case (0.33 [0.06] vs. 0.28 [0.08] (*p* = 0.0039)).

## Discussion

In this study, a map of the vibrotactile sense of the healthy upper limb was generated, seven males and three females. Neely and Burström (2006) reported, that no gender-specific differences are found during vibrotactile threshold measurements to the arm region, thus, we will not make any distinction between the gender of participants in the further discussion. The arm-shoulder region was divided into six dermatomes and three segments–the lower arm, the upper arm, and the shoulder region, each stimulated proximally and distally. For psychometric evaluation, tasks resulting in the quantification of sensation threshold, just noticeable difference, and perception of dynamically changing vibrotactile stimuli were used.

In our study, we decided to vary the amplitude of the stimulation, keeping the frequency fixed at 230 Hz. Due to the coupling effect between frequency and amplitude, while the frequency remains unaffected when the amplitude is modulated, the amplitude will change when the tactor operates outside the optimal frequency range, which is between 200–250 Hz for the C2 tactors (Engineering Acoustics Inc). Indeed, Dideriksen et al. (2021) shows that electrotactile stimulation performs better than C2 tactors at frequencies lower than 200 Hz. However, once the frequency of the vibrotactile approaches the optimal range, both stimulation modalities perform similarly.

### The sensation threshold

To be perceived, the stimulation applied to the shoulder segment needed to have a significantly higher vibration amplitude than the stimulation applied to the lower arm. The selected vibrotactile stimulation frequency (230 Hz) mainly targets the Pacini corpuscles in the skin (Saddik et al., 2011). The fact that the Pacini receptor density in the upper limb increases in the direction from the shoulder to the hand (Montagna, 1965; Johansson and Vallbo, 1979; Vallbo and Johansson, 1984), might explain our findings. As previously mentioned, we are not able to find any literature regarding differences in receptor density between the ventral and dorsal sides of the arm-shoulder region, as most of the studies investigate this only in the hand in which the dorsal side is covered in hairy skin and the ventral side in glabrous skin with their respective differences in innervation (Liu et al., 2007; Li et al., 2011). Since the arm is completely covered in hairy skin (Zimmerman et al., 2014) one would not expect such differences here. Interestingly, we find differences between the ST on the ventral and dorsal sides of the arm segments. These are significant only for the lower arm and the shoulder. In both segments, the ST is significantly lower ventrally. This might possibly be explained by the differential innervation of these locations. Another possible reason could be the thicker muscular tissue covering the bone on the dorsal parts of the lower arm and the shoulder, compared with their ventral counterparts. This proximity of the vibrotactile stimulation to the bone and the missing dampening of the soft tissue might lead to a better perception of the vibration. Indeed, Jacobs et al. (2000) performed an experiment in which they vibrotactilely stimulated the thumb/foot sole of the prosthetic and normal limb of amputees. They reported that upon vibratory stimulation of the residual limb, bone-anchored prostheses yielded better perception than socket prostheses, which are based on soft tissue support. Comparing the sensation threshold between the healthy hand and the socket prosthesis exposed an average increment of 20% for the affected side. When comparing the sensation threshold between healthy hands and bone-anchored prostheses, the affected side is similar to the control limb. This would also be consistent with the lack of significant differences in the upper arm in our study, where the ventral and dorsal muscle cover is similar.

Finally, all participants are able to correctly spatially identify the active vibrotactile unit. Given the spatial layout of tactile stimulation that is used in our experimental setup (see Figure 1B), it follows that vibrotactors can be successfully distinguished at a distance of 61 mm. This result is comparable with the reported ability of discrimination between two points (two-point discrimination) ranging from 30.7 to 42.4 mm (mid-posterior lower arm and lateral upper arm, respectively) (Nolan, 1982), suggesting that the perception of vibrotactile localization is in the same order of magnitude as two-point discrimination. Obviously, the lack of measurements at smaller distances leaves the possibility open that the subjects could correctly perceive distances between tactors below 61 mm.

### The Weber fraction and NDI

In contrast to the ST, we found no significant difference in any segment regarding either the Weber fraction (WF) or the number of discrete intervals (NDI). The same applies when comparing these outcomes ventrally vs. dorsally or observing the single segments (Figures 8, 9). These results are somewhat expected since the WF defines a *relative* change of amplitude between two stimuli, not an *absolute* one. In this case, it is the activation threshold of a single receptor (and to some extent, its signal to noise ratio) and not the density of receptors that determines the result. Therefore, assuming that we are activating the same family of receptors over the whole arm-shoulder region, the WF, and consequently, the NDI too, are expected to be similar in every location. In this light, our results suggest that we activated the same types of receptors at different sites of the investigated regions. Overall, previous studies on the perception of vibration on the lower arm show that the Weber fraction is in the range of 17% (at 200 Hz) to 30% (at 300 Hz) (Rothenberg et al., 1977; Mahns et al., 2006), which is consistent with our findings of a WF value of 20% across all locations in the arm-shoulder region at a 230 Hz stimulation frequency. Interestingly, the WF obtained on the fingertips using a similar setup and a frequency of 200 Hz was only ca. 18% lower than that of the lower arm, although the ST of the fingertip was ca. 63% below the value obtained on the lower arm (Mahns et al., 2006). This further supports the claim that the sensation threshold indeed decreases substantially more from the proximal to distal on the whole limb and that this is not the case for the Weber fraction.

Whereas, the sensation threshold represents a minimum *absolute* value of stimulation intensity that one can perceive, the Weber fraction measures, in contrast, a minimal *relative* change of stimulation intensity that an individual can detect. Therefore, while the ST can give some information about the receptor density this is not true for the WF, which reflects the receptors' overall physiological functioning and their interaction with the surrounding tissue. In the applied case of feedback reproduction in the healthy arm, it is possible to calculate the required difference between two stimuli at all points based on the measurement of the sensation threshold at the desired points, as well as the measurement of the Weber fraction at one of these points. This is possible if all regions have the same skin and similar structures. In fact, we tested this assumption by taking the ST at all measuring points and computing WF using the measured WF at one of these points. We repeated the procedure for all WFs and calculated a standard deviation of 0.31% with an absolute mean error of 0.44%. This error is about 2.2% of the mean measured Weber fraction and is therefore acceptable. This might help in the implementation of feedback, as the calibration would need to be performed once if the used frequency and amplitude are targeting one kind of receptor. The stimulation could then be provided over the whole arm with the same relative signal.

### The compensatory tracking task

There were no significant differences between the arm regions in the time delay that the subject exhibited in following the reference trajectory. This outcome could be potentially explained by the fact that the delay of the sensory pathway was relatively small in comparison with the other delays that were present in the control loop (e.g., the motor delay and cognitive processing delay), thus failing to account for a substantial difference across different arm-regions. Indeed, the mean distance from the stimulation site on the lower arm to the spinal cord was 63 cm, from the upper arm 43 cm, and from the shoulder 13 cm for our participants. The Pacinian corpuscles are innervated by Aβ (large, myelinated) fibers with conduction velocities up to 70 m/s (Montaño et al., 2010); this implies a travel time of 8.96 ms from the stimulation site of the lower arm, 6.15 ms from the upper arm, and 1.8 ms from the shoulder. These times lie in the interquartile range of the delay observed for each site and therefore do not account for differences in the time delay, thus correlating with our results. Therefore, the travel time of the stimuli, which is a consequence of nerve conduction velocity, do not significantly contribute to the measured time delay. This likewise suggests that the cognitive processing of the stimuli and the motor command execution delay are invariant to the alterations between the stimulation regions. Stepp et al. came to a similar conclusion investigating the importance of training compared with the importance of the vibration site. They found out that participants experienced a strong learning effect within a single session. The effects of the vibration site, however, are less dramatic (Stepp and Matsuoka, 2011).

Looking at the average rectified error, we found a difference between stimulated regions. In contrast to the the time delay, the rectified error is not dependent on the physiological reaction time but on the user's ability to correctly classify the provided feedback and properly react to it (see Figure 10B). Similar to the experimental task in which we calculated the WF, the subject could have been just differentiating between two consecutive stimuli and deciding which one was stronger. Consequently, a rectified error of zero would imply that the user would have been able to distinguish between infinite NDIs. As all regions possess the same NDIs, one might expect that no significant differences will be seen between the regions. However, the shoulder showed a significantly higher tracking error when compared with the upper arm. An explanation for this might be that the subject was using the aforementioned mechanism of comparing subsequent stimulations to determine the sign of the trend of the error (i.e., to determine if the error is increasing/decreasing), but here, (s)he also needed to know if the error is large or small (in absolute terms) in order to react accordingly. Therefore, the compensatory tracking task requires a combination of skills, that is, the ability of differentiating between *relative* changes and appropriately identifying the overall magnitude of the stimulation (i.e., its *absolute* value). This second aspect might contribute, just as it happens in the sensation threshold, to a deterioration of performance as one moves proximally. Indeed, the correlation coefficient indicates at least a statistical trend (corrected *p* < 0.1) of worsening performance between the lower arm and the shoulder.

### Practical implications for design of vibrotactile displays

Some of the results obtained during our experiment might have a significant impact on the design and evaluation of devices for vibrotactile stimulation of the upper limbs. For instance, we obtained significant differences between the sensation threshold of proximal and distal segments (Figures 4, 5). However, the perception of the relative changes in the stimulation intensity is invariant to the arm region (Figure 6). Moreover, the STs, although significantly different, are still very small with respect to the overall amplitude range–none of the measured locations exhibited an ST >6% of the maximal amplitude. One practical implication of these results is that the vibrotactors applied to the arm could be of similar size and power, independent of their location, facilitating their optimized mechanical design. This observation is further supported by the delays measured during the compensatory tracking task. Here, we discussed that the cognitive processing of the stimuli and the execution delay of motor commands are not significantly affected by the distance of the stimulation site to the spinal cord or its origin on the arm. Therefore, individuals are capable to use the feedback devices efficiently and with similar cognitive effort across all arm-shoulder dermatomes.

A specific application scenario could be vibrotactile feedback for upper-limb prostheses that communicates the prosthesis' grip force (or a similar variable) by modulating the vibration intensity (e.g., the higher the intensity the higher the grip force (Stephens-Fripp et al., 2018a)). In this context, our results are promising for amputees since the arm location on which feedback is delivered can significantly vary. This allows supplying individuals with different amputation levels with feedback (for transradial amputees on the lower arm, for transhumeral amputates on the upper arm or the shoulder). Given that the overall implementations of the feedback and prosthetic systems are similar, our results suggest that both transhumeral and transradial prosthetic users may have a similar level of proficiency in perceiving and interpreting the feedback (i.e., the prosthesis grip force). More specifically, since the WF was largely invariant between the upper and lower arm, both groups of individuals should be able to perceive and quantify the (relative) changes in grip force with similar performance (i.e., its relative increase or decrease from arbitrary nominal value). However, the lower ST of the lower arm also suggests that individuals suffering from transradial amputation could have advantages over their transhumeral counterparts in quantifying the actual amplitude of the prosthesis' grip force, i.e., in classifying the vibration intensity in absolute terms (e.g., as high, medium, or low). Nonetheless, the difference in the overall performance of the two subject groups is still unlikely to be functionally relevant: The tracking task has demonstrated remarkable similarity in the real-time interpretation of feedback across different arm-regions and every feedback interface is a part of an overarching sensory-motor integration framework. This framework consists of several intertwined layers, namely, the feedforward motor control, the control system, and the end-effector that, in combination with feedback, ultimately determine the outcome of the user's actions (Sensinger and Dosen, 2020).

Our data suggest that the receptors show a similar response to relative changes of the vibration stimuli, i.e., to those changes that are normalized to the perceivable range of stimulation—Weber fraction and number of distinct intervals are the same across all segments of the arm-shoulder region. However, different arm segments have different perceivable ranges of stimulation—the sensation threshold is significantly increasing from the distal to proximal regions. The higher tracking error in the tracking task in the shoulder compared with the arm region might have resulted from the smaller perceivable stimulation range in the shoulder region (i.e., the higher sensation threshold), leading to a decreased ability to properly assess the magnitude of the error in the tracking task. Nonetheless, even if some variations exist, the healthy arm and shoulder region can perceive vibrations at 230 Hz at relatively low amplitudes (in the range of 2–6%) and differentiate between two sequential stimulations if their amplitudes differ by 20%. As two of three outcome measures in the compensatory tracking task are invariant to a change in arm location, the ability of the subject to perceive dynamically changing stimuli is only marginally dependent on where it is applied.

Our discussion assumes that we are targeting the Pacini corpuscles. Even if the design of our study does not allow us to establish if there are differences between receptors since we do not correlate our results with histological studies, our data suggest that the receptors activated by the vibration stimuli show the same behavior across all locations, independent of their structural entity.

In summary, our experiments provide elementary insights regarding the vibrotactile sensation capacity of the healthy upper extremity. Since vibrotactile displays are the state-of-art in a wide array of applications, these results might contribute to an increased effectiveness of their use.

## Data availability statement

The raw data supporting the conclusions of this article will be made available by the authors, without undue reservation.

## Ethics statement

The studies involving human participants were reviewed and approved by the Clinical Ethics Committee of the University Medical Center Göttingen (Ethics Number: 26/6/20). The patients/participants provided their written informed consent to participate in this study.

## Author contributions

LP, MM, AS, and JE contributed to the conception and design of the study. LP and MM collected the data and wrote the first draft of the manuscript. LP and MAW performed the statistical analysis. All authors contributed to manuscript revision, read, and approved the submitted version.

## Funding

This study was partly supported by unrestricted grants of the German Federal Ministry of Education and Research BMBF to JE and AS (13GW0340B/16SV7657).

## Conflict of interest

The authors declare that the research was conducted in the absence of any commercial or financial relationships that could be construed as a potential conflict of interest.

## Publisher's note

All claims expressed in this article are solely those of the authors and do not necessarily represent those of their affiliated organizations, or those of the publisher, the editors and the reviewers. Any product that may be evaluated in this article, or claim that may be made by its manufacturer, is not guaranteed or endorsed by the publisher.
